# Correlation of Urine Loss after Catheter Removal and Early Continence in Men Undergoing Radical Prostatectomy

**DOI:** 10.3390/curroncol28060399

**Published:** 2021-11-15

**Authors:** Benedikt Hoeh, Felix Preisser, Mike Wenzel, Clara Humke, Clarissa Wittler, Jan L. Hohenhorst, Maja Volckmann-Wilde, Jens Köllermann, Thomas Steuber, Markus Graefen, Derya Tilki, Pierre I. Karakiewicz, Andreas Becker, Luis A. Kluth, Felix K. H. Chun, Philipp Mandel

**Affiliations:** 1 Department of Urology, University Hospital Frankfurt, Goethe University Frankfurt am Main, 60323 Frankfurt am Main, Germany; benedikt.hoeh@gmx.de (B.H.); mike.wenzel@kgu.de (M.W.); Clara.Humke@kgu.de (C.H.); clarissa.wittler@kgu.de (C.W.); M.Volckmann@gmx.de (M.V.-W.); andreas.becker@kgu.de (A.B.); luis.kluth@kgu.de (L.A.K.); felix.chun@kgu.de (F.K.H.C.); philipp.mandel@kgu.de (P.M.); 2 Cancer Prognostics and Health Outcomes Unit, Division of Urology, University of Montréal Health Center, Montréal, QC H3T 1C5, Canada; lukas@hohenhorst.com (J.L.H.); pierrekarakiewicz@gmail.com (P.I.K.); 3 Martini-Klinik Prostate Cancer Center, University Hospital Hamburg-Eppendorf, 20246 Hamburg, Germany; steuber@uke.de (T.S.); graefen@uke.de (M.G.); dtilki@me.com (D.T.); 4 Dr. Senckenberg Institute of Pathology, University Hospital Frankfurt, 60590 Frankfurt am Main, Germany; Jens.Koellermann@kgu.de; 5 Department of Urology, University Hospital Hamburg-Eppendorf, 20246 Hamburg, Germany

**Keywords:** urinary incontinence, radical prostatectomy, pad-test, incontinence, functional outcome

## Abstract

Background: To determine the correlation between urine loss in PAD-test after catheter removal, and early urinary continence (UC) in RP treated patients. Methods: Urine loss was measured by using a standardized, validated PAD-test within 24 h after removal of the transurethral catheter, and was grouped as a loss of <1, 1–10, 11–50, and >50 g of urine, respectively. Early UC (median: 3 months) was defined as the usage of no or one safety-pad. Uni- and multivariable logistic regression models tested the correlation between PAD-test results and early UC. Covariates consisted of age, BMI, nerve-sparing approach, prostate volume, and extraprostatic extension of tumor. Results: From 01/2018 to 03/2021, 100 patients undergoing RP with data available for a PAD-test and early UC were retrospectively identified. Ultimately, 24%, 47%, 15%, and 14% of patients had a loss of urine <1 g, 1–10 g, 11–50 g, and >50 g in PAD-test, respectively. Additionally, 59% of patients reported to be continent. In multivariable logistic regression models, urine loss in PAD-test predicted early UC (OR: 0.21 vs. 0.09 vs. 0.03; for urine loss 1–10 g vs. 11–50 g vs. >50 g, Ref: <1 g; all *p* < 0.05). Conclusions: Urine loss after catheter removal strongly correlated with early continence as well as a severity in urinary incontinence.

## 1. Introduction

With an estimated incidence of 1.3 million cases of newly-diagnosed cases in 2018, Prostate cancer (PCa) ranks as the second most frequent cancer worldwide, accounting for approximately 15% of all cancers worldwide [1,2]. While radical prostatectomy (RP) can provide favorable cancer control in both localized and locally advanced stage disease, ensuring suitable functional outcomes represents a central issue after radical prostatectomy [3,4,5,6,7]. Among those, postoperative urinary incontinence has been reported to have a far-reaching negative impact on patients’ quality of life, and represents a potential bothersome side-effect [4,8,9]. Recently, Ilie et al. reported a meaningful association between urinary incontinence and increased mental distress (odds ratio [OR] = 4.79) in a contemporary cohort of PCa patients treated with RP, highlighting the worrisome impact urinary incontinence can have on the quality of life in PCa patients [10]. Substantial research has been conducted to elucidate potential risk factors, such as age, body mass index (BMI), and the experience of surgeons for the postoperative urinary incontinence in patients undergoing RP with primary end-points of interest at 3 and 12 months [3,11,12,13,14]. However, current data is scarce about easily operable and reliable tools to predict early continence rates at a very timely point of convalescence.

We addressed this void by relying on a standardized, validated instrument, namely the PAD-test, to measure the urine loss within 24 h after a transurethral catheter removal. We hypothesized that urine loss (defined in the PAD-test) after catheter removal was correlated with early urinary continence rates and thus, could be used to identify patients at the highest-risk of postoperative urinary incontinence at a very early-on timepoint. We tested this hypothesis in a contemporary cohort of 100 PCa patients being treated with RP at a tertiary referral center.

## 2. Material and Methods

### 2.1. Study Population

From 01/2018 to 03/2021, 664 patients treated with RP were retrospectively identified from the prospective institutional database of the University Hospital Frankfurt. Of those, 100 patients (15.1%) were subsequently identified with data available for a PAD-test, as well as early continence follow-up assessments. Indication for RP was histologically confirmed prostate cancer. From 01/2020 ongoing, PAD-test measurements were scaled back to ensure a minimum-stay to prevent COVID-19 transmission [15]. The study was approved by the institutional review boards (ethical approval: SUG-1-2018) of the University Cancer Centre Frankfurt and the Ethical Committee at the University Hospital Frankfurt. All patients included in our study signed a written informed consent.

All surgeons, who performed RP in the current cohort, were experienced surgeons trained in high-volume prostate cancer centers. RP was routinely performed with full functional-length urethral sphincter (FFLU) and neurovascular bundle preservation (NVBP) with intraoperative frozen section technique (IFT), as previously described [16,17,18,19,20].

### 2.2. Outcome Measurements

**Figure 1:**
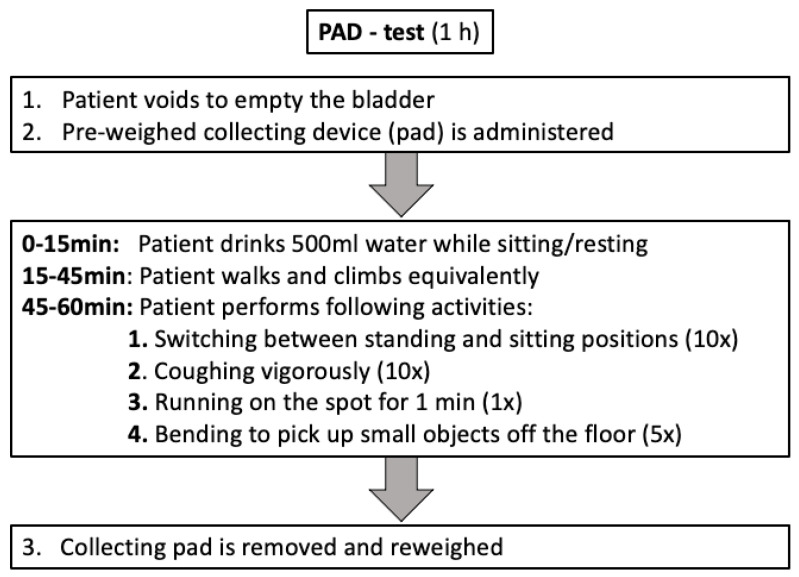
Flowchart depicting the sequence of the PAD-test.

Data regarding perioperative and early continence was ascertained by PAD-test results and the usage of pads in follow-up assessments after RP. The PAD-test was a comprehensible and validated test that measured the amount of involuntary loss of urine while performing predefined physical activities within 1 h. The PAD-test was performed within 24 h after the removal of the transurethral catheter, as previously described [17,21]. Urine loss of <1 g, 1–10 g, 11–50 g, and >50 g was defined as continent, mild incontinent, moderate incontinent, and severe incontinent, respectively (Figure 1). Early continence was defined as the use of no, or one safety-pad within 24 h, whereas a higher number of pads was considered incontinent. Early continence status was based on a voluntary self-reported standardized, established questionnaire [4]. More precisely, data regarding daily pad usage was assessed by evaluating the number of pads used, grouped as ’0–one safety’, ‘1–2’, ‘3–5’, or ‘>5’ pads, respectively. If two follow-up assessments were available within the first six months of post-surgery (*n* = 3), the more mature assessment (closer to 6 months cut-off) was considered for further analyses.

### 2.3. Statistical Analyses

Descriptive statistics included frequencies and proportions for categorical variables. Medians and interquartile ranges (IQR) were reported for continuously coded variables. The chi-square test examined the statistical significance of the differences in proportions, while the Kruskal-Wallis test was used to examine differences in medians.

Uni- and multivariable logistic regression models tested the relationship between urine loss after catheter removal in PAD-tests (<1 g vs. 1–10 g vs. 11–50 g vs. >50 g) and early urinary continence (0–1 vs. ≤1 pads/24 h). Covariates consisted of age at RP (≤60 vs. 61–69 vs. ≥70 years), BMI (<25 vs. 25–30 vs. >30 kg/m^2^), prostate volume (≤40 vs. >40 mL), extraprostatic extension of tumor (pT2 vs. pT3/4), and nerve-sparing approach (no vs. yes).

To test for a potential underlying selection bias, sensitivity analyses were performed between the current study cohort and patients with missing data regarding PAD-test results and early continence rates in the study period (01/2018 to 03/2021).

For all statistical analyses, R software environment for statistical computing and graphics (version 3.4.3) was used [22]. All tests were two-sided with a level of significance set at *p* < 0.05.

## 3. Results

### 3.1. Descriptive Characteristics of the Study Population

In total, 100 patients were included in the current analysis (Table 1). Of those, 74 patients (74%) underwent robotic-assisted RP, whereas 26 patients underwent (26%) open RP, respectively. The median age was 65 years (IQR: 58–59), the median PSA was 8 ng/mL (IQR: 6–12) and the median BMI was 26.1 kg/m^2^ (IQR: 24.3–29.9). Final histopathological examination revealed in 45% an extraprostatic extension of the tumor. Nerve sparing approach (uni/bilateral) was performed in most cases (93%), and median operation time was 218 min (IQR: 189–252).

### 3.2. Perioperative and Early Continence Outcomes

PAD-test following catheter removal recorded 24%, 47%, 15%, and 14% of patients having a loss of urine <1 g (continent), 1–10 g (mild incontinent), 11–50 g (moderate incontinent), and >50 g (severe incontinent), respectively. In early follow-up assessments (median: 3 months; IQR: 2–5 months), 59% of patients were continent, defined by the usage of no or one safety-pad within 24 h. Tabulation, according to PAD-test result, exhibited 88%, 62%, 40%, and 21% urinary continence in patients with loss of urine <1 g, 1–10 g, 11–50 g, and >50 g (Table 2).

### 3.3. Uni-and Multivariable Logistic Regression Models

In univariable logistic regression models, urine loss in the PAD-test was a statistically significant factor that influenced urinary continence in early assessments, and resulted in an odds ratio of 0.23 [95%-CI: 0.05–0.79; *p* = 0.03], 0.10 [95%-CI: 0.02–0.43; *p* = 0.004], and 0.04 [95%-CI: 0.01–0.20; *p* < 0.001] for 1–10 g, 11–50 g, and >50 g urine loss, respectively (Table 3). Besides age ≥70 years, which was associated with a lower chance of continence [OR: 0.28; 95%-CI: 0.08–0.87; *p* = 0.03], neither BMI, extraprostatic extension, prostate volume, nor nerve-sparing approach were significant risk factors in univariable analyses. After adjustment in multivariable logistic regression models, higher urine loss remained to be a factor lowering the chance for early continence (Urine loss of 1–10 g, 11–50 g, and >50 g resulted in an odds ratio of 0.21 [95%-CI: 0.04–0.79; *p* = 0.03], 0.09 [95%-CI: 0.01–0.48; *p* = 0.008], and 0.03 [95%-CI: 0.004–0.18; *p* < 0.001]). All other variables had an insignificant influence on early urinary continence in multivariable analyses.

### 3.4. Sample Selection Bias

Sensitivity analyses were performed for potential selection bias, due to differences in tumor and patient characteristics between the study cohort (*n* = 100) and patients with missing data regarding PAD-test results or early continence rates in the study period (*n* = 564). Here, no significant differences between the current study cohort and the entire cohort (all *p* ≥ 0.1) were recorded.

## 4. Discussion

The preservation of continence after RP is a crucial aspect in the treatment of patients with PCa [1]. Several studies have demonstrated that postoperative urinary incontinence after RP can result in a substantial reduction in the patients’ quality of life, and represents a potential bothersome side-effect [4,8,9]. To date, data regarding reliable measurements to predict continence rates at a very timely point of convalescence are rare. We hypothesized that urine loss (defined in a PAD-test) after catheter removal was correlated with early continence rates and thus, could be used to identify patients at the highest-risk of postoperative urinary incontinence and as such, a higher need of intensified, postoperative pelvic floor training. We relied on a contemporary cohort of 100 PCa patients being treated with RP at a tertiary referral center and made noteworthy findings.

First, PAD-test results strongly correlated with continence status in the early follow-up assessments. Patients considered to be continent according to the PAD-test results (<1 g urine loss) reported an early continence rate of 88% in follow-up assessments, whereas rates for mild incontinent (1–10 g urine loss) and moderate incontinent (11–50 g urine loss) patients were 62% and 40%. Least frequent, yet the most severe incontinence in PAD-tests (≥50 g urine loss) resulted in low rates of early continence (22%). This correlation can also be seen in multivariable logistic regressions for all subgroups after adjusting for further risk factors of early postoperative incontinence. A urine loss, e.g., of >50 g, resulted in a strikingly decreased chance of early urinary continence (odds ratio: 0.03) compared to patients with urine loss <1 g. The current literature is scarce about the relationship between continence status after catheter removal and early continence in RP treated patients [23,24]. For example, Manfredi et al., even though ascertaining urinary continence after RP at different time points beginning with catheter removal, solely relied on the number of pads as a proxy for urinary continence throughout their study [24]. Therefore, the study by Manfredi et al. could not be directly compared to the current study, since pad usage represented a fairly inaccurate measurement tool, and might not represent the full bandwidth of urinary incontinence at such an early timepoint [24]. Contrary to Manfredi et al., Ates et al. relied on a more precise variable, namely the urine loss ratio (ULR), to predict early, mid-term, and long-term continence rate of PCa patients undergoing laparoscopic RP [25,26]. Urine loss ratio was defined as the weight of urine loss in the pad divided by daily micturition volume (24 h). Even though the authors were able to find a cut off 0.15 ULR, above which the level of incontinence increased in a manifold fashion, the ULR represented a labor- and time-consuming variable to harbor in everyday clinical practice, since its protocol relied on an extended collective time span of 24 h. Regarding the reduced length of stays following RP in the current era, it was questionable if such an extensive test can be implemented in routine clinical practice. By contrast, the current introduced PAD-test could be seen as a timesaving (2 vs. 24 h) measurement tool and was additionally less labor intensive. Consequently, to the best of our knowledge, the current study was the first to report a robust correlation between urine loss after catheter removal and early continence rates relying on a time-efficient, reproducible, and robust measurement tool, namely PAD-test.

Second, urine loss in the PAD-test strongly correlated with the severity of early incontinence. Instead of dichotomic ascertainment of urinary continence after catheter removal, stratifying the severity of incontinence into predefined categories, made a more precise correlation possible. For example, patients with a loss of more than 50 g in PAD-test were at the highest risk (28.6%) for serious early incontinence (>5 pads/24 h), compared to patients with urine loss of 11–50 g (6.7%) or 1–10 g (0%) in a PAD-test. The same correlation trends were recorded for less profound early incontinence (3–5 pads/24 h). From a clinical point of view, current findings may be attributable to a potential malfunction of the external sphincter; either pre-existing or by an injury of the urethral sphincter during RP [18,27]. Even though full functional-length of urethral sphincter preservation was routinely performed in all patients, interindividual anatomical and tumor characteristics may have influenced the extent of preservation, and led to potential injury of the sphincter [28]. As a consequence, PAD-test results did not only profoundly correlate with early continence rate, but could also be taken as a measurement tool to estimate the severity of early incontinence after RP. Interestingly, preliminary data reveal clear trends that solely patients with severe urinary loss of >50 g in PAD-tests fail to regain full continence recovery in long-term follow-up (>12 months). Even though the primary focus of the current study was to investigate the correlation of PAD-test results with early continence rates, the findings added to the picture that pad-test results were of great value to estimate the severity of continence, even at a longer time of follow-up. These findings were in accordance with several previous studies, which have demonstrated a prolonged recovery time for urinary continence beyond 12 months following RP [29,30].

Finally, other variables, such as BMI, age, and prostate volume, did not meet a level of significance in the multivariable logistic regression models for early continence [31]. This could be explained by certain risk factors (e.g., age) for urinary incontinence that might simultaneously account as risk factors for PAD-test results. Theissen et al. reported that younger patients showed significantly better early continence rates relying on PAD-test results after catheter removal compared to their older counterparts [17]. Consequently, a lack of significance in the current study could be explained due to these observations.

The current study was not devoid of limitations. First and foremost, were the limitations inherent to the retrospective nature of the study and the limited sample size. Second, the population in the current study underwent open and robotic-assisted radical prostatectomy, and the differences in experience among the surgeons might be present. However, it is of note that all surgeons underwent training in high-volume prostate cancer centers. Third, since no routine bladder neck reconstruction was performed in the current study population, comparison of continence results to patients undergoing RP with bladder neck reconstruction should be interpreted accordingly [32]. Fourth and finally, a potential bias regarding the extent of postsurgical pelvic-floor training cannot be ruled out. All patients were strongly encouraged to seek professional pelvic-floor training for urinary continence recovery and were also instructed during their in-patient stay.

## 5. Conclusions

Urine loss after catheter removal strongly correlated with early urinary continence and could be used to estimate the severity of urinary incontinence. Therefore, PAD-test after catheter removal may identify men with a higher need of intensified, postoperative pelvic floor training. Additional studies may elucidate the correlation between PAD-test results and long-term continence rates in the future.

## Figures and Tables

**Table 1:** Patient and clinicopathological characteristics of 100 patients treated with radical prostatectomy at the University Hospital Frankfurt between 01/2018 and 03/2021, with data available for both PAD-test and early continence status. All values are median (IQR) or frequencies (%).

	Study Cohort, (*n* = 100)
Age in years, Median (IQR)	65 (58, 69)
PSA in mg/mL, Median (IQR)	8 (6, 12)
Body Mass Index in kg/m^2^, Median (IQR)	26.1 (24.3, 29.9)
International Prostate Symptom Score, Median (IQR)	6.5 (3, 9)
Body Mass Index grouped in kg/m^2^, *n* (%)	
≤25	33 (34%)
25–30	40 (41%)
≥30	25 (26%)
D’Amico risk classification, *n* (%)	
low	13 (13%)
intermediate	57 (57%)
high	30 (30%)
Surgical approach, *n* (%)	
robotic-assisted RP	74 (74%)
open RP	26 (26%)
Operation time in min, Median (IQR)	218 (189, 252)
Prostate volume in cm^3^, Median (IQR)	40 (30, 50)
pT-stage, *n* (%)	
pT2a	6 (6.0%)
pT2b	1 (1.0%)
pT2c	48 (48%)
pT3a	33 (33%)
pT3b	10 (10%)
pT4	2 (2.0%)
Extraprostatic extension of tumor, *n* (%)	
no	55 (55%)
yes	45 (45%)
pN-stage, *n* (%)	
pN0	85 (85%)
pN1	4 (4.0%)
pNx	11 (11%)
cM-stage, *n* (%)	
M0	96 (96%)
M1	4 (4.0%)
Gleason Grade Group RP-specimen, *n* (%)	
I	9 (9%)
II	53 (54%)
III	19 (19%)
IV	4 (4%)
V	13 (13%)
Nerve sparing, *n* (%)	
none	7 (7%)
uni/bilateral	93 (93%)
Positive surgical margin, *n* (%)	
R0	63 (63%)
R1	34 (34%)
Rx	3 (3%)

**Table 2:** Usage of pads in early continence follow-up assessments in 100 patients treated with radical prostatectomy between 01/2018 and 10/2020 at the University Hospital Frankfurt, stratified according to urine loss in PAD-test; All values are frequencies (%).

		0–1(Safety) Pad/24 h	1–2 Pads/24 h	3–5 Pads/24 h	>5 Pads/24 h
Urine loss in g, *n* (%)					
<1 g	24 (24.0%)	21 (87.5%)	2 (8.3%)	1 (4.2%)	0 (0%)
1–10 g	47 (47.0%)	29 (61.7%)	12 (25.6%)	6 (12.7%)	0 (0%)
11–50 g	15 (15.0%)	6 (40.0%)	6 (40.0%)	2 (13.3%)	1 (6.7%)
>50 g	14 (14.0%)	3 (21.4%)	5 (35.7%)	2 (14.3%)	4 (28.6%)

**Table 3:** Uni- and multivariable logistic regression models predicting early urinary continence in 100 patients treated with radical prostatectomy between 01/2018 and 10/2020 at the University Hospital Frankfurt. Urinary continence was defined as the usage of no or one safety pad within 24 h. Extraprostatic extension was defined by pT3/pT4 in final RP-specimen stage.

	Logistic Regression Models							
	Univariable				Multivariable			
	Odds Ratio	95%-CI		*p*-Value	Odds Ratio	95%-CI		*p*-Value
PAD-test urine loss in g								
<1	Ref.				Ref.			
1–10	0.23	0.05	0.79	0.03	0.21	0.04	0.79	0.03
11–50	0.10	0.02	0.43	0.004	0.09	0.01	0.48	0.008
>50	0.04	0.01	0.20	<0.001	0.03	0.004	0.18	<0.001
Age in years								
≤60	Ref.				Ref.			
61–69	0.49	0.17	1.30	0.16	0.42	0.13	1.28	0.14
≥70	0.28	0.08	0.87	0.03	0.55	0.14	2.15	0.39
Nerve-sparing approach								
No	Ref.				Ref.			
Yes	3.96	0.81	28.67	0.11	1.52	0.23	13.51	0.68
Body Mass Index kg/m^2^								
<25	Ref.				Ref.			
25–30	1.55	0.60	4.02	0.36	2.04	0.66	6.56	0.22
≥30	1.06	0.37	3.05	0.91	1.06	0.29	3.86	0.93
Extraprostatic Extension								
No	Ref.				Ref.			
Yes	0.77	0.34	1.72	0.53	1.29	0.46	3.71	0.63
Prostate volume in mL								
≤40	Ref.				Ref.			
>40	1.11	0.50	2.51	0.80	0.82	0.30	2.20	0.69

## Data Availability

The data presented in this study are available on request from the corresponding author.
